# Cross-cultural adaptation of body image assessment instruments for university students: a systematic review

**DOI:** 10.1186/s41155-021-00177-w

**Published:** 2021-04-19

**Authors:** Ravine Carvalho Pessanha Coelho da Silva, Ana Carolina Soares Amaral, Augusta Karla Silva Quintanilha, Vitor Alexandre Rabelo de Almeida, Marcus Vinicius Freitas Rodrigues, Aldair J Oliveira, Fabiane Frota da Rocha Morgado

**Affiliations:** 1 Federal Rural University of Rio de Janeiro, Rio de Janeiro, Brazil; 2 Rio de Janeiro, Brazil; 3 Federal Institute of Education, Science and Technology of Southeast of Minas Gerais, Campus Barbacena, Barbacena, Brazil

**Keywords:** Translation, Adaptation, Methodology, Validity, Reliability, Young

## Abstract

The theoretical-methodological process of cross-cultural adaptation of scales is fundamental for the expansion of research possibilities related to a given area and population, as instrument adequacy allows for the consistent assessment of the construct being studied. This article aims to highlight the different theoretical-methodological processes of cross-cultural adaptations of scales for assessing body image among young university students. The articles were selected in February 2020, with the following search descriptors: (“body image”) and (young or “college students” or graduating or graduat *) and (“cross-cultural validation” or “cross-cultural adaptation”), in three databases—Scopus, PsycINFO, and Web of Science, without any time restrictions. Altogether, 304 studies were surveyed on the different bases mentioned above, and 14 studies published between 2010 and 2019 were evaluated. This study highlighted the methodological procedures used in the process of cross-cultural adaptation, as well as the population, the types of validity and reliability, and also the main limitations, strengths, and results reported in each study included in this review. The information presented in this review will allow new researchers to make more appropriate choices about instruments for assessing body image in young university students, in addition to presenting the methodological evolution on this theme.

**Supplementary Information:**

The online version contains supplementary material available at 10.1186/s41155-021-00177-w.

## Introduction

Body image is the mental representation that one has of their own body (Schilder, 1999). It can be characterized by cognitive, affective, and behavioral components (Cash & Pruzinsky, 2002; Slade, 1994) and is constantly influenced by social, libidinal, and other aspects (Cash & Pruzinsky, 2002). Many researchers have striven to understand the construct of body image by investigating the aspects related to its development and its constant influences on different phases of life (Laus et al., 2014).

University students have been shown to be vulnerable to different alterations to body image. Studies reveal that university students report high body dissatisfaction and unhealthy practices related to body weight maintenance (Claumann et al., 2017; De Souza & Alvarenga, 2016; Frank et al., 2016). Moreover, university students are considered to be at risk for developing depression, low self-esteem, diminished wellbeing, interpersonal difficulties, suicidal ideation, and eating and body dysmorphic disorders (Barra, Silva, Maroco, & Campos, 2019; de Carvalho et al., 2013; De Souza & Alvarenga, 2016; Sarhan, Krey, Chaud, & Abreu, 2015; Schaefer et al., 2015). Thus, these matters deeply impact the health, professional formation, and the whole academic journey of these university students (Behmani & Kumar, 2016; Felden et al., 2016; Ponte, Fonseca, Carvalhal, & da Fonseca, 2019).

Systematic investigations concerning this population may contribute to tracking risk groups and promoting intervention strategies on body image by creating a healthier and more positive relationship between students and their bodies, therefore preventing the appearance of many comorbidities (Guimarães, Aquino, Prado, & Rodrigues, 2020). To achieve these goals, instruments for assessing body image and valid and reliable measures of the various components of the body image construct must be provided.

One of the main strategies for obtaining psychometric instruments is through the process of cross-cultural adaptation, a series of rigorous and cautious methodological procedures which ensure that the instrument remains equivalent to the original version created in another language and/or culture. It, therefore, allows one scale created for a specific context to be used in another population (Beaton, Bombardier, Guillemin, & Ferraz, 2000; Swami & Barron, 2019). It is recommended that a methodological guide is used for this process, one which describes in detail the methodological procedures to be adopted.

Numerous studies have aimed to perform a cross-cultural adaptation of body image assessment instruments, especially for young university students (Swami & Barron, 2019). It can be said that knowing the main theoretical/methodological findings of these studies, as well as the main limitations pointed out by the authors in the area, can contribute to improving the quality of future studies. Therefore, this research aimed to highlight the different theoretical-methodological processes of cross-cultural adaptations of scales for assessing body image among young university students.

## Methods

This systematic review was duly registered at PROSPERO, under the registration number CRD42020145182, and followed the recommendations proposed by the Preferred Reporting Items for Systematic Reviews and Meta-analysis: The Prisma Statement (Moher et al., 2009). The Prisma Statement checklist can be viewed in the supplementary material in this review article.

Articles were selected from three databases: Scopus, Web of Science, and PsycINFO. There was no time restriction for the literature search, which was completed in February 2020. The following search terms were used, only in the English language: (“body image”) and (Young or “College Students” or graduating or graduat*) and (“cross-cultural validation” or “cross-cultural adaptation”). The selection process in the databases was carried out by two independent researchers. In case of disagreement in selecting studies, a third researcher was consulted. Descriptive analysis was used for data analysis, as well as the categorical content analysis of Bardin (1977). Content analysis was used to group information from studies included into similar topics, in other words, into categories. It was also used to make inferences from these results feasible.

The selection of articles was performed initially by reading the titles of all articles found. Those studies in which the search terms were present in the title were included in the first selection. After the first selection from the title, the summary was read in full for more detailed information about the study. Studies that presented information about systematic reviews in the summary were included, and studies that did not have this topic were excluded. After including the articles according to their titles and abstract, the selected articles were read in full to verify if these studies met the inclusion criteria of this systematic review. The articles whose themes were not in agreement with this systematic review’s objective were definitively excluded. After this procedure, the final number of studies included in this review was reached.

As criteria for the inclusion of articles, we used (1) articles whose objective was to describe the cross-cultural adaptation of scales for assessing body image and (2) studies carried out with a population of young university students. The following were excluded from this systematic review: (1) articles in languages other than English, Portuguese, and Spanish; (2) articles that did not highlight the objective of carrying out the cross-cultural adaptation of scales; (3) articles that, despite having this objective, did not have the theme of body image; and (4) articles that addressed the methodological process in question, but were developed in populations other than university students.

### Quality assessment and data extraction

To assess the quality of the studies, the Quality Assessment Tools (QATSDD) (Sirriyeh, Lawton, Gardner, & Armitage, 2012) were used. The data for evaluation were extracted directly from the articles included in this systematic review. The QATSDD consists of 16 items for quality assessment, 14 of which are applied to qualitative and quantitative studies, and two of which are used for mixed methods. Each study was scored on a scale from zero to three points in each item, with the minimum score being equal to zero when the authors of each study did not mention the information highlighted in each category; equal to one point when the authors mentioned very little information; equal to two points when the information was made somewhat available in the study; and a maximum score of three points when the information was presented in full. The score was subjective and individual. It is noteworthy that all studies included in this review are quantitative. The quality of each analyzed study was calculated from the percentage of the maximum quality achieved, with a maximum possible score of 42 points. Articles with a score equal to or greater than 21 points (a quality rating of 50%) were classified as having good or high quality. In contrast, studies that did not reach this score were considered to be of lower quality than expected (Sirriyeh et al., 2012).

## Results

**Fig. 1:**
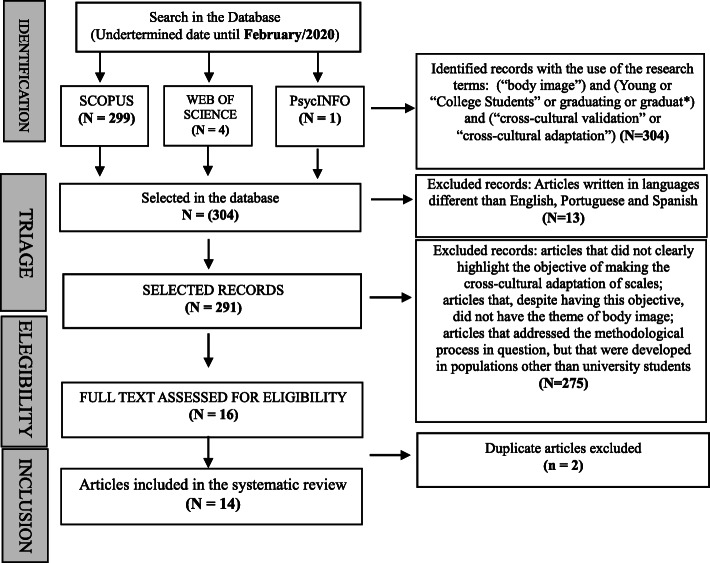
Flowchart presenting the review of the systematic process of identification and selection of articles. Source: The author, 2021

This systematic review evaluated 14 studies. Figure 1 presents a flowchart that summarizes the process of searching for and selecting the articles found, as well as the process of including and excluding studies after individual analysis of all titles, abstracts, and subsequently, all texts in full.

The included articles were analyzed using the following categories as reference: authorship, publication year, place of development, objective, guideline used for the process of cross-cultural adaptation of scales, sample of each study, type of validity and reliability assessed, main limitations and strengths reported by the authors, and additionally, the main results found in each included study. These detailed results can be seen in Table 1.

**Table 1:** Detailed results: Studies included in this systematic review

	Author Year Location	Objective (To assess the cross-cultural adaptation of the…)	Methodological guide	Population	Type of validity	Type of reliability	Reported limitations	Reported strengths	Main results	Quality (Value / %)
**01**	Barra et al. (2019) Brazil	SATAQ-4 to the Portuguese Language and Brazilian students	Beaton, Bombardier, Guillemin, and Ferraz (2007)	1051 YUS—BS 18–30 years old	CFA FV / CV CTTV / DV / CVV	IC	Dificulty in the generalization of results.	V and R of the SATAQ-4	*λ* = 0.729–0.976; *χ*^2^/gl = 8.39; CFI = 0.98; TLI = 0.98; RMSEA = 0.08; *r* = 0.063–0.590; α = 0.84–0.95.	40 / 95.2
**02**	Shoji, Mehling, Hautzinger, and Herbert (2018) Japan	MAIA to the Japanese population	Beaton et al. (2000)	390 YUS—BS mean age of 20.3 years old	EFA CV / CTTV / DV / DVV / CVV	IC	Cultural differences affected Differences in the sample Reduction of 7 items in the scale	Difference in the factorial structure	λ > 0.40; α = 0.67–0.87	33/78.5
**03**	Chakroun-Baggioni, Corman, Spada, Caselli, and Gierski (2017) France	DTQ to a sample of university students	Brislin, 1986	436 YUS—BS mean age of 19.1 years old	CFA PV	IC / TRT	Measures SR Cross-sectional model (makes it difficult to draw conclusion of causality)	Study related with different measures	λ > 0.50; SB scaled *χ*^2^ (34)= 150.60, *p* <0.001, SB scaled *χ*^2^ /df=4.43; RMSEA=0.09 (90% IC: 0.07–0.10); CFI=0.94. ICC= 0.71; 95% CI = 0.50–0.83 Omega coefficients= 0.84–0.90	37 / 88.0
**04**	Bas et al. (2017) Turkey	IES-2 to the Turkish language	Brislin (1986) Bracken and Barona (1991)	377 YUS—BS 19–31 years old	EFA CVV / DV / CTTV	IC/ TRT	SH CS Results cannot be compared to other groups	Good V and R R same as the original study EQ between scales	*λ* =0.69–0.96, KMO =0.87, *χ*^2^ = 9043.49 (p < 0.001), *α* = 0.82.	37 / 88.0
**05**	Ulian et al. (2017) Brazil	FCQ-S and FCQ-T to the Portuguese language.	Reichenheim and Moraes (2007)	22 YUS—W 20–24 years	CV	IC	SH Inaccuracy of the original translation	Excellent verbal comprehension Advances in research	*α* = 0.5–0.8	32 / 76.1
**06**	Carbonneau et al. (2016) Canada	IES-2 to Canada	Beaton et al. (2000)	334 W and 75 M 18–65 years old	CFA CTTV / DV	IC / TRT	Sample specificity SH	SH Wide age range	*λ* =0.51 – 0.93, *χ*^2^ (df = 222, *n* = 239) = 479.97, *p* < 0.001; NNFI = 0.911; CFI = 0.922; RMSEA = 0.070; 90% CI of the RMSEA = 0.061–0.078. *r* = 0.64 to 0.90, α >0.70.	38 / 90.4
**07**	Silva, Costa, Pimenta, Maroco, and Campos (2016) Brazil	BSQ to use in Brazil and Portugal in female university students	It is unclear	278—Portugal 248—Brazil YUS averagely 18 years old	CFA CVV / CCV	IC	It is unclear	It is unclear	*λ* =0.34-0.87, *χ*^2^ /df 2.65–5.63, CFA and CFI = 0.81–0.95, NFI=0.78–0.93, RMSEA=0.07–0.09, α=0.88–0.97.	32 / 76.1
**08**	Rousseau, Denieul, Lentillon, and Valls (2014) France	MBDS to France	Guillemin, Bombardier, and Beaton (1993)	319 M YUS 15–23 years old	EFA and CFA CCV	IC / TRT	It is unclear	MBDS could be a useful instrument in identifying and detecting problems linked to body image in men	*λ* >0.40, *χ*^2^ [300] = 3381.13, *p* < 0.001, KM0 = 0.90, Chi2/df = 3.20; AGFI = 0.82; GFI = 0.86; RMSEA = 0.08, α=0.82–0.88.	33 / 78.5
**09**	Pakpour, Zeidi, Ziaeiha, and Burri (2014) Switzerland	FGSIS-I – Iran Version - in a sample of college women	Guillemin, Bombardier, and Beaton (1993); Beaton et al (2000)	1877 W YUS 19–29 years old	EFA and CFA CVV/ CTTV / FV / CV / DV	IC/ TRT	Cross-sectional project SR Non-confidential data CS / SH	Instrument highly V e R	λ = 0.45–0.83 KMO =0.78 and a *χ*^2^ = 3649.05, df= 21, *p* < 0.001. *χ*^2^= 153.93, df= 14, *p* = 0.00004, GFI=0.86, NFI = 0.84, CFI = 0.85, and RMSEA = 0.191; α=0.79–0.86.	30 / 71.4
**10**	Campana, Tavares, Swami, and da Silva (2013) Brazil	DMS, SMAQ and MBIDS to Brazilian Portuguese	Beaton, Bombardier, Guillemin, and Ferraz (2002)	878 YUS—M 18–39 years old	CFA CTTV / DV / CV	IC	The scales were validated for a specific group of Brazilian men, instead of a larger and more heterogeneous group (e.g., adolescents and older adults)	Useful tools for investigations in body image Decrease of cultural barriers	λ >0.30, DMS Factor Structure = *χ*^2^ = 239.28, *p* < .001; RMSEA = .067, GFI = .992, AGFI = .986, NFI = .987, CFI = .992, NNFI = .989, *χ*^2^/gl = 4.98), *α* =0.86–0.87. SMAQ Factor Structure = *χ*^2^ = 266.62, *p* < .001; RMSEA = .050, GFI = .993, AGFI = .990, NFI = .990, CH = .996, NNFI = .995, *χ*^2^/df = 3.17, α =0.64–0.90. MBIDS Factor Structure = *χ*^2^ = 27.51, *p* < .001; RMSEA = .053, GFI = .998, AGFI = .995, NH = .996, CFI = .999, NNFI = .999, *χ*^2^/df = 3.43, α =0.84.	38 / 90.4
**11**	de Carvalho et al. (2013) Brazil	MBDS to male students in Brazil	Herdman, Fox-Rushby, and Badia (1998)	59 YUS—BS mean age of 23.5 years old	CV	IC	Needs psychometric analysis of validity and reliability	Advances in research	α = 0.92	21 / 50.0
**12**	Conti et al. (2012) Brazil	BCI to the Portuguese language	Reichenheim and Moraes (2007)	47 YUS—BS mean age of: 22.7 years old	CV	It is unclear	Absence of V and R	Easy verbal comprehension	It is unclear	28 / 66.6
**13**	Amaral, Cordás, Conti, and Ferreira (2011) Brazil	SATAQ-3 to the Brazilian Portuguese language.	Guillemin, Bombardier, Beaton (1993)	146 YUS—70 M (mean age of 20.7) and 76 W (mean age of 20.3).	CV	IC	The article described only the initial cross-cultural adaptation process of the SATAQ-3. Absence of the V and R.	Items with easy comprehension. Items were not excluded IC similar to the original study	*α* = 0.74–0.92	34 / 80.9
**14**	Conti, Scagliusi, Queiroz, Hearst, and Cordás (2010) Brazil	TIS to the Portuguese language	Pasquali (2000) Herdman et al. (1998)	108 YUS—51 M (mean age of 18 ) 57 W (mean age of 19)	CV	IC	It is unclear	Items with easy comprehension. Satisfactory scores in the V	α > 0.80	21 / 50.0

Source: Articles included in this systematic review

*Abbreviations: AGFI* Adjusted goodness-of-fit index, *BCI* Body Change Inventory, *BS* both sexes, *BSQ* Body Shape Questionnaire, *CCV* concurrent validity, *CFA* confirmatory analysis, *CFI* Comparative Fit Index, *CS* convenience sampling, *CTTV* construct validity, *CV* content validity, *CVV* convergent validity, *PV* predictive validity, *DMS* Drive for Muscularity Scale, *DTQ* Desire Thinking Questionnaire, *DV* discriminant validity, *DVV* divergent validity, *FCQ-S* State Food Cravings Questionnaires, *FCQ-T* Trait Food Cravings Questionnaires, *FGSIS-I* Female Genital Self-Image Scale, *FV* face validity, *GFI* Goodness-of-Fit Index, *IC* internal consistency, *ICC* intraclass correlation coefficient, *IES-2* Intuitive Eating Scale 2, *KMO* Kaiser-Meyer-Olkin index, *M* men, *MAIA* Multidimensional Assessment of Interoceptive Awareness, *MBDS* Male Body Dissatisfaction Scale, *MBIDS* Male Body Ideal Distress Scale, *NFI* Normed Fit Index, *NNFI* Non-normed fit index, *R* Reliability, *RMSEA* Root Mean Square Error of Approximation, *SATAQ-3* Sociocultural Attitudes Towards Appearance Questionnaire-3, *SATAQ-4* Sociocultural Attitudes Towards Appearance Scale-4, *SH* sample homogeneity, *SMAQ* Swansea Muscularity Attitudes Questionnaire, *TIS* Tripartide Influence Scale, *TRT* test-retest, *V* validity, *W* women, *YUS* young university students, *α* Cronbach’s alpha, *λ* factor loading, *χ*^2^ Bartlett’s test of sphericity, *χ*^*2*^*/gl* chi-square test

**Fig. 2:**
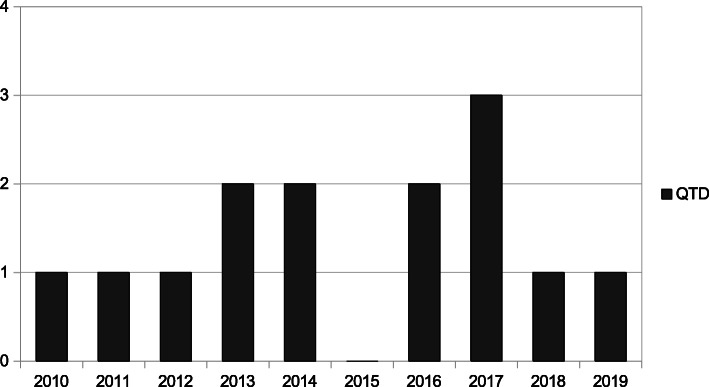
Chronological evolution of the studies included in this review. Source: The author, 2020. *N*, number of articles included

In the analysis performed on the included studies, it was observed that they were all published between 2010 and 2019. Half of the studies were published in the last 5 years (*N*=7; 50%), with 2017 as the year with the most publications on this issue (*N*=3; 21.4%). The chronological evolution of the published articles is shown in Fig. 2.

Based on the articles included in this review, Brazil was the country with the largest number of studies (*N*=8; 57.1%), followed by France (*N*=2; 14.2%), and then by Japan, Turkey, Canada, and Switzerland with 1 study each (7.1 %).

The most widely used set of guidelines for cross-cultural adaptation was the one proposed by Beaton et al. (2000, 2002, 2007), which served as basic reference for five (35.7%) studies included in this review. Table 2 shows all methodological guidelines used in the analyzed studies.

**Table 2:** Methodological guidelines used in the studies included in this systematic review

Methodological guide	***N***	%
Beaton et al. (2000, 2002, 2007)	5	35.7
Guillemin, Bombardier, Beaton (1993)	2	14.2
Brislin (1970, 1986)	2	14.2
Reichenheim and Moraes (2007)	2	14.2
Herdman et al. (1998)	2	14.2
Pasquali (1999)	1	7.1
Bracken and Barona (1991)	1	7.1
It is unclear	1	7.1

Source: The author, 2020

*N* number of studies

Regarding the instruments for body image assessment that were put under the process of cross-cultural adaptation, the majority appeared in only one study, excluding the instruments: (1) Intuitive Eating Scale-2 (IES-2) and (2) Male Body Dissatisfaction Scale (MBDS). Most studies were carried out with sample populations that included both sexes (*N*=10; 71.4%). Two studies counted only with women (14.2%) and two studies had only men as the target population (14.2%).

Regarding the types of validity evaluated, it is important to highlight that, in the studies included, content validity, confirmatory factor analysis, construct validity, and discriminant validity were more frequent, with 64.2%, 50%, 42.8%, and 42.8%, respectively. As for reliability, the internal consistency measure was the most expressive in the studies, as seen in almost all articles (*N* = 13; 92.8%); the test-retest measure represented the second most used measure (*N* = 5; 35.7%). More detailed information can be seen in Table 3.

**Table 3:** Types of validity and reliability used in the studies included in this systematic review

Type of validity	***N***	%	Type of reliability	***N***	%
Content	9	64.2	Internal consistency	13	92.8
Confirmatory factor analysis	7	50	Test-retest	5	35.7
Construct	6	42.8	Intraclass correlation	1	5.5
Discriminant	6	42.8	Reproducibility	1	5.5
Convergent	5	35.7	Stability	1	5.5
Exploratory factor analysis	4	28.5	It is unclear	3	16.6
Face	2	14.2			
Concurrent	2	14.2			
Divergent	1	7.1			
Predictive validity	1	7.2			
Unclear	1	7.2			

Source: The author, 2020

*N* number of studies

Furthermore, when focusing on the main study limitations reported by the authors, sample homogeneity stands out, shown in two (14.2%) of the analyzed studies. Articles that do not present this information, a total of three (21.4%), and all other studies included in this systematic review have different limitations. In addition, concerning the strengths of the studies, good validity and reliability can be highlighted, in addition to a good understanding of the items in four studies (28.7%), respectively.

The detailed description of these results is shown in Table 4.

**Table 4:** Distribution of limitations and strengths reported in the studies included in this systematic review

Limitations	***N***	%	Strengths	***N***	%
Sample homogeneity	4	28.7	Valid and reliable instrument	4	28.7
Absence of psychometric analysis	1	7.1	Excellent verbal comprehension of items	4	28.7
Absence of validity and reliability	1	7.1	Advances in research	2	14.2
Cross-sectional data collection	1	7.1	Same reliability as the original study	2	14.2
The initial process of cross-cultural adaptation	1	7.1	Decrease of cultural barriers	1	7.1
Testing in other populations	1	7.1	Utile and potent tool which allows rapid triage	1	7.1
Cultural differences	1	7.1	Difference of factorial stability	1	7.1
Loss or addition of items	1	7.1	Utilization of different measures	1	7.1
Non-confidential data	1	7.1	Equivalent scales	1	7.1
Translation inaccuracies	1	7.1	Wide age range	1	7.1
Absence of comparison between groups	1	7.1	Items were not lost	1	7.1
Difficulty in the generalization of results	1	7.1	Identify and detected body Image problems	1	7.1
Not informed (it is unclear)	3	21.4	Sample heterogeneity	1	7.1
			Not informed (it is unclear)	1	7.1

Source: The author, 2020

*N n*umber of studies

Concerning the main results, we highlight two points: (1) results of the factorial structure of the instruments and (2) internal consistency analysis.

In regard to the first aspect, all studies analyzed had a minimum factor loading in the confirmatory factor analysis (CFA) and in the exploratory factor analysis (EFA), as recommended in the literature, λ> 0.30 (Hair Júnior et al., 2009). The reference values in three of the four studies that carried out EFA were also adequate—Kaiser-Meyer-Olkin (KMO)> 0.6 and Bartlett’s test of sphericity (*χ*^2^) ≤ 0.05 (Hair Júnior et al., 2009). One of the studies that carried EFA did not make these values clear (Shoji et al., 2018), making analysis impossible. In CFA, the reference values are *χ*^2^/gl = chi-square test = ideal <3 and acceptable between 3 and 5. AGFI (Adjusted Goodness-of-Fit Index), NFI (Normed fit index), NNFI (Non -normed Fit Index), CFI (Comparative Fit Index), and GFI (Goodness-of-Fit Index) > 0.90. RMSEA (Root Mean Square Error of Approximation) < 0.08 (Hair Júnior et al., 2009). Although these values are recognized in the literature, some studies did not achieve them. For example, Barra et al. (2019) presented *χ*^2^/gl = 8.39; Rousseau et al. (2014) reported the following AGFI values = 0.82; GFI = 0.86, and Pakpour et al. (2014) highlighted GFI = 0.86, NFI = 0.84, CFI = 0.85. RMSEA values that did not fit the reference standard were also found, as example RMSEA = 0.191 (Pakpour et al., 2014) and RMSEA = 0.09 (Chakroun-Baggioni et al., 2017). It is worth mentioning that only two studies (14.2%) combined the use of CFA and EFA (Pakpour et al., 2014; Rousseau et al., 2014). And yet five studies (35.7%) did not perform any type of analysis of the factorial structure of the instruments (Amaral et al., 2011; Conti et al., 2012; Conti et al., 2010; de Carvalho et al., 2013; Ulian et al., 2017).

Regarding the analysis of internal consistency, 12 studies (85.7%) used *α* = Cronbach’s alpha (α), one study used Omega coefficients (Chakroun-Baggioni et al., 2017), and one study did not make it clear the type of reliability adopted (Conti et al., 2012). Minimum reference values for internal consistency are considered above 0.6 (Malhotra, 2004). In the studies included in this review, all the internal consistency values were above the minimum limit recommended in the literature, which is expressed by the range of 0.64-0.97.

Lastly, concerning the quality of the studies, it is evident that 12 of them (85.7%) had scores above the pre-established average of 50%, which indicates that these articles were of good quality (Amaral et al., 2011; Barra et al., 2019; Bas et al., 2017; Campana et al., 2013; Carbonneau et al., 2016; Chakroun-Baggioni et al., 2017; Conti et al., 2012; Pakpour et al., 2014; Rousseau et al., 2014; Shoji et al., 2018; Silva et al., 2016; Ulian et al., 2017). As for the remaining percentage, two articles (14.2%) scored exactly the previously determined average, indicating acceptable quality (Conti et al., 2010; de Carvalho et al., 2013). No article presented below-average quality, which would be an indicator of poor quality.

Regarding the studies included in this systematic review and their quality, although none presented below-average quality, some studies did not present good quality. However, we have chosen to consider them, regardless of the score achieved. Studies that do not have an expected quality can likewise serve as a reference for future research because they can serve as guidelines for developing future quality studies.

## Discussion

This systematic review aimed to identify the current practices of cross-cultural adaptation of body image assessment scales for young university students. The results showed a variety of practices, which will be discussed below along with recommendations for future investigations on the topic.

Analyzing the studies included in this review, we found that the process of cross-cultural adaptation of body image scales for young university students was first recorded in 2010, followed by an increase in studies over the next few years, with peaks in publications in the years 2013 and 2014, 2016 and 2017, bringing together the largest amount of research.

Another point to be highlighted in this review concerns the locations where the studies were carried out. Brazil appears to be the main place for carrying out this type of research, with 8 out of the 14 studies selected in this review being developed in this country. This finding allows the inference of two counterpoints. On the one hand, Brazil appears to be a powerful example of quantitative reference in the researchers’ instrumentalization through the process of cross-cultural adaptation for the assessment of body image among young university students. This can enable the expansion of cross-cultural studies carried out in the country.

On the other hand, it is possible to state that Brazilian researchers may be too much focused on “importing” instruments created in other countries and perhaps not as well involved in the creation of new assessment scales specific to the Brazilian population and cultural context. Although it is not possible with this study to make a comparison of cross-culturally adapted instruments created in Brazil, there is a great involvement of national researchers in the cross-cultural adaptation of instruments, when compared to researchers from other countries. Morgado et al. (2014) consider that the creation of new measures is recommended when there are no instruments already created and validated correctly in other countries. Perhaps, this justifies the fact that Brazil opts to carry out cross-cultural adaptation of instruments, rather than the creation of them. Possibly, on account of the fact that the international literature has shown an abundance of instruments aimed to evaluate different facets of university students’ body image. Additionally, cross-cultural adaptations enable multicultural studies, in the sense that data comparison among different groups becomes possible (Carvalho, Amaral, & Ferreira, 2014), which might be another justifying reason why Brazil leads the ranking of cross-cultural adaptations.

In this regard, Campana and Tavares (2009) add that Brazil has a lack of instruments about body image, and making a cross-cultural adaptation is important to expand the possibilities of research in our country. These notes and Brazil’s growing interest in researching body image justify Brazil’s leadership in translation, cross-cultural adaptation and validation of already existing instruments in the literature.

Additionally, we observed that Beaton’s guide (Beaton et al., 2000; 2002; 2007) was the most used methodological guide for cross-cultural adaptations of instruments, reported in previous studies as useful and usual in the scale adaptation procedure (Argyrides, Kkeli, & Kendeou, 2014; Swami & Barron, 2019). It describes a cultural adaptation model in medical, sociological, and psychological literature, which is a complete adaptation process that includes translation alongside semantic, idiomatic, and experiential adaptations, as well as the conceptual equivalence between the original instruments and their adaptations (Hendricson et al., 1989; Swami & Barron, 2019; Swami et al., 2019).

Another used guide was that of Guillemin et al. (1993), which was based on previous studies in psychology and sociology. It differs from Beaton’s guide as it also recommends, if relevant, the re-examination of the questionnaire scores beyond the proposed steps. Moreover, two other methodological guides appeared in three studies selected in this review. Both follow the same proposal as the other five guides already highlighted above, and therefore, they will not be detailed. Among the studies gathered in this review, one did not clarify which guides it used in the methodological process. However, it is widely recommended that a methodological guide be used to perform the cross-cultural adaptation of instruments in the area of body image (Swami & Barron, 2019), in order to enable the maintenance of cultural, semantic, idiomatic, conceptual, and experimental equivalences in the adapted instrument (Guillemin, 1995).

Nevertheless, Swami and Barron (2019) argue that the best practices indicate that a combination of translation procedures should be adopted as there is no consensus in the literature about a single guide to be used. According to these authors, while Beaton et al. (2000) provides a structure that several body image scholars will find useful due to its clarity in the steps of the methodological process (Argyrides et al., 2014; Carbonneau et al., 2016; Swami et al., 2019), this structure also requires time and effort, which can lead some researchers to view combined translation techniques as less demanding alternatives in the process of cross-cultural adaptation (Swami & Barron, 2019).

Among the 14 studies included in this review, different psychometric instruments were chosen to be translated and adapted through the cross-cultural adaptation process. These instruments have been commonly used to assess the following components of body image, or associated aspects: sociocultural attitudes related to appearance (Amaral et al., 2011; Barra et al., 2019; Conti et al., 2010); thoughts and desires related to body image (Chakroun-Baggioni et al., 2017; Shoji et al., 2018); body dissatisfaction or feelings directed towards muscularity (Campana et al., 2013; de Carvalho et al., 2013; Rousseau et al., 2014); eating attitudes and behaviors (Bas et al., 2017; Carbonneau et al., 2016; Ulian et al., 2017); dissatisfaction with weight and body shape (Silva et al., 2016); sexual activity (Pakpour et al., 2014); and body change (Conti et al., 2012).

At this point, an interest in the multiple dimensions of the body image held by university students is observed. This can be justified by the fact that body image is a multifaceted and complex construct, requiring different instruments to fully assess. It is also noteworthy that body image is an essential phenomenon in several aspects of human life, ranging from biological aspects related to health and diseases, to psychosocial aspects such as quality of life (Campana & Tavares, 2009; Cash & Pruzinsky, 2002; Thompson, 2004).

Among the two instruments cited as the target of more than one study, the results showed good evidence of psychometric qualities. The Intuitive Eating Scale-2 (Bas et al., 2017; Carbonneau et al., 2016), adapted for Turkey and Canada, assesses the tendency of individuals to follow their tracks of hunger and satiety related to when and how much to eat. The instrument showed good validity and reliability, confirming the psychometric properties of the original study. The Male Body Dissatisfaction Scale (de Carvalho et al., 2013; Rousseau et al., 2014) was adapted for France and Brazil to assess body dissatisfaction towards muscularity. Its internal consistency has been proven, demonstrating the instrument’s good reliability.

The study that evaluated the Tripartite Influence Scale describes different stages of the cross-cultural adaptation of the scale. Conti et al. (2010) performed the translation, back-translation, and assessment of the verbal comprehension of the instrument. Similarly, the study by Amaral et al. (2011) described the stages of adaptation of the Sociocultural Attitudes Towards Appearance Questionnaire-3 to the Portuguese language. Both studies, as well as others identified (Conti et al., 2012; de Carvalho et al., 2013; Ulian et al., 2017), evaluated only the content validity of the instruments. This can be a limitation since the safe use of measurement instruments for a given population is directly related to the assessment of its psychometric qualities. It is worth noting that some of the adapted instruments in the aforementioned studies continued in future studies that assessed their psychometric qualities; this was the case with the Sociocultural Attitudes Towards Appearance Questionnaire-3 (Amaral et al., 2011), from the Tripartite Influence Scale (Amaral et al., 2011), and the Male Body Dissatisfaction Scale (Carvalho et al., 2015).

Furthermore, most of the studies selected in this systematic review recruited a sample of both sexes (*N* = 10), while two studies were conducted only with women, and the other two studies were conducted strictly with men. This may indicate greater sample heterogeneity, which is considered adequate in the processes of cross-cultural adaptation as it often presents different results and possible new findings, thus representing more reliable results (Barra et al., 2019; Dahl, Wickman, & Wengström, 2014). However, some authors argue that sample heterogeneity can represent a very large diversification of results, which does not represent the totality that is expected to be measured. According to these authors, this can hinder the consensus of the results (Bas et al., 2017; Ferreira, Corazza, Francisco, & Neves, 2018; Swami et al., 2019). Therefore, future studies should consider the pros and cons of recruiting homogeneous or heterogeneous samples, with regard to population sex. These findings are in line with what has been pointed out in previous literature, which has found that, especially in the last two decades, both men and women are dissatisfied with their bodies (de Carvalho & Ferreira, 2014; Hobza & Rochlen, 2009; Kelley, Neufeld, & Musher-Eizenman, 2010), which justifies the availability of instruments for both sexes. Therefore, it is necessary to choose appropriate body image assessment instruments for each of these groups, investigating the specificities of the construct for men and women. It also justifies the use of the psychometric processes of scales for both men and women (Beaton et al., 2000; Gardner & Brown, 2010; Thompson, 2004).

Regarding the assessment of validity, this psychometric quality determines whether, in fact, the test measures what it is supposed to measure (Pasquali, 2009). Barra et al. (2019), Bas et al. (2017), and Pakpour et al. (2014) pointed out in their studies the strength of the instrument to be considered valid and reliable, based on the aforementioned validities, assessed in the study. Among the main validities developed in these studies are the content validity, CFA, construct, and discriminant validities. It is worth noting that CFA (50%) was used more than EFA (28.5%). This can be justified by the fact that the factorial structure of the instruments that have undergone the process of cross-cultural adaptation has already been tested in other countries. Then, the CFA would serve to confirm or refute the previous factorial structure. A similar previous review study concerning the development of scales found different results, with EFA being more recurrent than CFA (Morgado et al., 2014). In comparison with this previous study, we can infer that the process of creating a new instrument requires exploring the new factorial structure, which justifies the EFA. Studies with cross-cultural adaptation demand to confirm or refute previous factorial models. This fact justifies greater use of CFA in the context here investigated.

Reliability is considered one of the main quality assurance criteria of the instrument, as it refers to the ability of the test to measure without errors (Pasquali, 2009; Primi, 2012). This criterion focuses on obtaining consistent and reproducible results when measuring a given attribute (Fayers & Machin, 2007). The most used reliability tests in the studies were internal consistency and test-retest. These tests are related to the coherence, consistency of the results, and the confidence that the test inspires in measuring the phenomena without large fluctuations between repeated measures, which would reflect the presence of measurement errors. In other words, these procedures estimate the level of accuracy of a test and establish an expectation of how erroneous the measurement can be (Kimberlin & Winterstein, 2008; Martins, 2006; Pasquali, 2009; Primi, 2012). Therefore, imprecise or incomplete psychometric procedures, performed in this methodological process, will probably bias the results (Morgado et al., 2014). At this point, it is highlighted that specifically, one study did not specify, or did not clearly show, which reliability method it used (Conti et al., 2012).

Regarding the limitations, three studies did not make this information clear in the text. Therefore, it is possible to highlight a diversity of specific limitations highlighted in each included study. The only limitation that is repeated in the studies (*n* = 4, 28.7%) is related to the characteristics of the sample, highlighting the homogeneity of the sample because the results cannot be reproduced and generalized to other groups (Bas et al., 2017; Carbonneau et al., 2016; Pakpour et al., 2014; Ulian et al., 2017).

Moreover, the following limitations also stand out: (a) lack of validity and reliability. However, several authors emphasize that validity and reliability are psychometric attributes that must be present in a good instrument and must be considered in studies of psychometric adaptation, since their absence can generate errors in the statistical conclusions of the research and can be considered a limitation of the developed study (Cunha, de Almeida Neto, & Stackfleth, 2016; Pilatti, Pedroso, & Gutierrez, 2010; Swami & Barron, 2019), and (b) the cross-sectional model used in the studies, which does not allow the establishment of cause and effect relationship, representing a certain vulnerability to the simultaneity bias. However, although this does not allow a definition of the temporal sequence between the events studied, we can point out positive points of this methodology, highlighting that it is a method widely used in studies which examine the relationship between events, being simple, low cost, and objective in data collection (Chakroun-Baggioni et al., 2017; Gonçalves & Silvany, 2013).

When we observe the main strengths described in the included studies, the following stand out: (a) the instruments are valid and reliable (Barra et al., 2019; Bas et al., 2017; Conti et al., 2010); (b) they have excellent verbal comprehension (Amaral et al., 2011; Conti et al., 2010, 2012; Ulian et al., 2017); and (c) they promote advances in research (Campana et al., 2013; de Carvalho et al., 2013; Ulian et al., 2017). We can infer, from the results, that researchers recognize the importance of psychometric qualities and also a meticulous semantic adaptation process as outstanding aspects in their studies, together with the recommended procedures for carrying out the process of cross-cultural adaptation (Beaton et al., 2000; Swami & Barron, 2019).

Although several authors recognize the importance of the psychometric qualities of scales, we were able to observe flaws in this process when analyzing the statistical results of the factorial structure of instruments. For example, some studies (35.7%) did not do any type of factorial analysis of the instruments. Others used only EFA or CFA. Among those that performed EFA, most studies presented adjusted reference values. However, one study made no mention of such values at all. Among those who did CFA, some studies presented inadequate reference values, according to what is recommended in the literature, and this can be considered a problem because it weakens the instrument's validation process. Additionally, only two studies combined EFA and CFA. For more consistent results on the psychometric indices of a scale, Morgado et al. (2014) indicate the combined use of EFA and CFA. Most of the studies included in this review are in the opposite direction of the abovementioned recommendation.

Regarding internal consistency, which is the most widely used measure of reliability (Morgado et al., 2014), only one study failed to present this value. Among the others, they all presented values that represent what is recommended in the literature. This measure was the reference in the studies that described it.

In relation to the quality of the articles included, it can be noted that the studies with the lowest scores were the oldest (date of publication between 2010 and 2013). The concentration of studies with low quality in this period may be linked to the increase of studies in the area after 2016, which may have had better support for research through previous studies, greater methodological rigor, as well as advances in research related to the constructs in question. It is important to note, therefore, the importance of publishing the limitations and difficulties found in the studies, in order to further improvements in the development of future research and prevent the same errors from being made by new researchers.

It is also noteworthy that the articles with high-quality scores presented an explicit methodological rigor, as well as a detailed presentation of the information about their research processes. This might offer a better understanding of the study for potential readers, as well as better reliability of the information about the research.

This review has some limitations that must be considered. Initially, when selecting articles, studies may not have been included due to the search terms used, which may have impacted the results. Furthermore, another limitation that needs to be highlighted is the fact that the initial inclusion of the studies was made based on the title. This may have contributed to the loss of studies on cross-cultural adaptation since the title does not always represent the entire content of an article. Finally, our study is only current until February 2020. Studies published after this period could not be included in this review.

Despite these limitations, this study presents important contributions. First, this systematic review analyzed consistent information about the literature in question, updated until February 2020, making the article up to date. In addition, this review presents both the evolution of studies on body image among university students, as well as a broad discussion on this theme, permeating the different constructs that encompass body image. Thus, it is expected to assist future researchers in the knowledge and choice of body image assessment instruments for the public in question.

## Conclusion

This systematic review presents different studies that have been cross-culturally adapted to other populations. In all of them, the theoretical-methodological procedures chosen by the authors to perform the cross-cultural adaptations were analyzed, such as, for example, the instruments, the methodological guides, as well as the types of validity and reliability used. Several concepts and methodological strategies, as well as the limitations and successes of different studies, were presented and discussed as an overview for future research on the topic. Thus, we believe that this article presents important contributions to the scientific literature, mainly because it provides a comprehensive set of information that can increase the quality of future research practices in cross-cultural adaptation of body image instruments.

## Supplementary Information

**Additional file 1.** PRISMA checklist.

## Data Availability

All data from the articles included in this review are presented in a properly organized table and were submitted in supplementary material.
