# Correction to: Cross-cultural adaptation of body image assessment instruments for university students: a systematic review

**DOI:** 10.1186/s41155-021-00180-1

**Published:** 2021-05-18

**Authors:** Ravine Carvalho Pessanha Coelho da Silva, Ana Carolina Soares Amaral, Augusta Karla Silva Quintanilha, Vitor Alexandre Rabelo de Almeida, Marcus Vinicius Freitas Rodrigues, Aldair J. Oliveira, Fabiane Frota da Rocha Morgado

**Affiliations:** 1 Federal Rural University of Rio de Janeiro, Rio de Janeiro, Brazil; 2 Rio de Janeiro, Brazil; 3 Federal Institute of Education, Science and Technology of Southeast of Minas Gerais, Campus Barbacena, Barbacena, Brazil

**Correction to: Psicol Refl Crít 34, 11 (2021)**

**https://doi.org/10.1186/s41155-021-00177-w**

Following publication of the original article (da Silva et al., 2021), the authors identified an error in the author name of Fabiane Frota da Rocha Morgado.

The incorrect author name is: Fabriane Frota da Rocha Morgado.

The correct author name is: Fabiane Frota da Rocha Morgado.

The author group has been updated above and the original article (da Silva et al., 2021) has been corrected.

## References

[CR1] da Silva RCPC (2021). Cross-cultural adaptation of body image assessment instruments for university students: a systematic review. Psicologia: Reflexão e Crítica.

